# Dog filariosis in the Lazio region (Central Italy): first report on the presence of *Dirofilaria repens*

**DOI:** 10.1186/1471-2334-5-75

**Published:** 2005-09-26

**Authors:** Paola Scaramozzino, Simona Gabrielli, Michele Di Paolo, Marcello Sala, Francesco Scholl, Gabriella Cancrini

**Affiliations:** 1 Istituto Zooprofilattico Sperimentale delle Regioni Lazio e Toscana, Via Appia Nuova 1411, 00178 Rome, Italy; 2 Parasitology Section, Department of Public Health Science, University of Rome 'La Sapienza' Piazza Aldo Moro, Rome, Italy

## Abstract

**Background:**

Epidemiological investigations were carried out in the Lazio Region to assess the status of canine filariosis and to evaluate the actual risk for veterinary and medical public health.

**Methods:**

Since August 2001 to June 2003, a total of 972 canine blood samples, collected in public kennels and from private owners animals of the 5 Provinces of the Region, were tested. The presence of filarial parasites was evaluated by microscopy and bio-molecular techniques; the species identification was performed by means of the same diagnostic tools.

**Results:**

A total of 17/972 (1.75%; 95%CI 1.06%–2.85%) blood samples were parasitized by *D. repens*,13 out them drawn by dogs resident in the Province of Roma, and 4 in the other provinces. Multivariate analysis was performed in order to evaluate the association between filariosis and risk factors. The origin from coastal territories seems to be a significant risk factor to acquire the infection.

**Conclusion:**

This is the first report of canine filariosis in the Lazio Region, where *D. repens *was before reported only in foxes. The risk of human zoonotic infection is stressed, and the absence of other filarial species is discussed

## Background

Filarial nematodes described in dogs are: *Dirofilaria immitis, D. repens, Acanthocheilonema reconditum, A. dracunculoides *and *Cercopitifilaria grassi *(Order: Spirurida, Superfamily: Filarioidea, Family: Onchocercidae). The most prevalent species are *D. immitis, D. repens*, and *A. reconditum*, that show a different geographical distribution: cosmopolitan for *A. reconditum *and *D. immitis*, restricted to the Europe, Middle East, Asia and Africa for *D. repens. D. immitis *is responsible for heartworm disease, whereas the other species produce subcutaneous or splanchnic infections. Furthermore, in areas where dog filarioses are endemic, at least *D. immitis *and *D. repens *are recognized as etiological agent of zoonotic infections in humans.

Canine heartworm disease is regarded as one of the most dangerous threat for the dog health, but it is an emerging sanitary problem also for cats. This dirofilariosis is endemic-hyperendemic in the Northern Italy (the Po Valley is the largest endemic area), with prevalence rates ranging from 22 to 68% (even 80% where animals are not receiving chemoprophylaxis) [1-5]. Similar high prevalence is reported from other countries in Southern Europe [6,7], where the incidence rate observed during the last decades has been increasing, with a northward spread of the infection [5]. This trend is confirmed, in Italy, by recent surveys that found endemic some previously disease-free areas [4]. Different is the pattern of heartworm infection in Central and Southern Italy, where much lower infection rates (13%) [8] are observed, or its presence is reported only occasionally [9,10]. As far the Lazio region, up to now there have been no reports about autochtonous infections, despite the presence of competent vectors belonging to the *Culex *and *Aedes *genera.

*D. repens *is considered scarcely pathogenic and therefore its distribution is less studied. In Europe the parasite has been reported in Bulgary (1%) [11], in Switzerland (1.6%) [12], in Greece (12–37%) [13], in France (1.36%) [14] and in the mediterranean side of Spain with infection rates ranging from 5.1% to 84.6% [15,16]. As far as Italy the species is reported with increasing prevalence from northern to southern regions. The parasite has been evidenced recently in most regions of central Italy (Toscana, Umbria, and Campania) with prevalences ranging from 2 to 21%. In detail infection rates reported for canine subcutaneous dirofilariosis in aformentioned regions are 21.1%; 6% and 2% respectively [17,8,10]. In the Lazio region *D. repens *has been reported only in foxes in the 60ies [18]; since then, no additional data on its geographical distribution and its presence among the dog population are available. The present study is, therefore, aimed to assess the status of canine filariosis in this region, considering in particular the public health risk in the city of Rome, where the relationship between dog and human populations is extremely tight. The role of some potential risk factors has been also investigated.

## Methods

### Study area

The Lazio Region is 17,207 km large and its territory is divided among 5 Provinces: Roma, Viterbo, Rieti, Latina and Frosinone. It is bordered by the Tyrrhenian Sea to the West and by the Apennine mountains (ca. 2,000 m a.s.l.) to the East. Landscape is mainly hilly, with coastal plains only taking about 20% of the territory. Some of the pre-existing natural marshy lands have been dried at the time of anti-malaria campaigns. Climate is classified as Mediterranean or sub-tropical, with dry summer and mild winters.

There are no official data about the canine population, but it is estimated to be 400,000 only in the Province of Roma, about 200.000 of which in the capital.

### Sampling protocol

Since August 2001 to June 2003 a sampling protocol on canine population of the Lazio Region was carried out in two phases. In the first sampling phase, aimed to test the dog population of the Roma Province, two sub-samples were defined: (i.) ownership animals (n = 320) and (ii.) dogs from 6 public kennels (n = 352). The former (i.) was calculated to single out at least one positive at a 95% confidence level, assuming 1% of expected prevalence. In order to define a strategy to sample ownership dogs sera, the city was subdivided in six study areas, lying both in the urban internal territory (n = 4) and along the coast (n = 2). For each area, one private veterinary practice was involved in the survey. Veterinarians were asked to bleed dogs randomly chosen among the ones brought to clinics for routinely control and were also asked to collect basic information data. The second sub-sample (ii.) was obtained by a convenience sampling of a variable number (15–91) of dogs from each kennel.

Only animals older than 6 month, not recently treated by antihelmintics, have been included in the study. Dogs were bled before 9:00 in the morning or after 5:00 in the afternoon, to assure sampling times that fit in with the microfilariae circadian rhythm.

In the second phase, in order to extend the study and to acquire some more information on the distribution of canine filariosis in the entire Region, a supplementary convenience sampling was performed in four kennels, each located in one of the remaining 4 Provinces of the Lazio, and each keeping dogs caught in the one's territory. In every one of the kennels, which were lacking of insect proof nets, 7 to 97 dogs were tested.

Data about age, sex, hair size (short/long), living habitat (kennel/private house), province of residence, area of residence (urban/rural, coast/hill/mountain) for each dog were collected and computerized.

Further samples (about 170) coming from routinely diagnostic activity of the Istituto Zooprofilattico Sperimentale di Roma were included in the final data set.

Altogether, a total of 972 dog blood samples were tested in this study.

Filarial infection was detected by microscopic and bio-molecular techniques.

### Microscopic analysis

EDTA blood samples were kept at 4°C until microscopic examination, performed usually within 2–3 days. Blood was processed according to the modified Knott method

[19]. Microfilariae were looked for at low magnification (200X) and then observed at 400X and 1000X in order to identify the species on the basis of morphological features [20]. Samples were then stored at -20°C for the successive analysis.

### Bio-molecular analysis

Samples positive to microscopy were examined individually, to confirm species specificity of microscopic identification, whereas negative samples were examined on pools of 4 samples each. DNA extraction was achieved by the QIAamp *DNA blood extraction kit *(Qiagen). A first PCR-based analysis was performed according to the protocol previously developed [21], using "filarial" specific ribosomal primers named S2-S16 [22]. The amplification give rise to a product of about 400 bp for most filarial species, whereas *D. repens *yields an additional fragment of about 350 bp. Amplification products were excised from agarose gel, purified with the Nucleo Spin *® *Extract kit (Macherey-Nagel), and analysed for sequencing by M-Medical Srl. Sequences comparison was achieved by CLUSTAL analysis [23]. Moreover, PCR positive samples were further tested using primers specific for *D. repens *and *D. immitis *[21], to confirm the identification of filarial species. Amplification products expected are of 325-bp and a ladder consisting of multiples of 175-bp for *D. repens*, and a minor 348-bp fragment with a major 747-bp fragment for *D. immitis*.

### Data analysis

The prevalence of filariosis and the confidence interval of the estimates at 95%confidence level, based on the results of microscopic and molecular analysis, were calculated for the whole sample and for each Province of the dog origin. Only for the dog population coming from the Roma Province, whose data collection procedures were well controlled, more accurate and uniformly distributed on the territory, the complete data set was available. Univariate logistic linear regression analysis at 95% confidence level was performed in order to estimate the effect of each of the following variables on the risk of filariosis: distance from the coastline (<20 Km; >20 Km); urbanization level of the origin area (rural/urban); breeding in kennel/private house; dog age (6–12; 13–36; 37–120; > 120 months); hair length. To investigate the possibility of confounding in the observedunivariate associations, we conducted multivariate logistic regressions with all candidate independent variables. The Odds Ratios (OR) and the 95% confidence intervals (95%CI) were calculated. A *P*-value of <0.05 was considered significant. All analyses were conducted using SPSS^® ^10.0 (SPSS Inc., 1999).

## Results

Among the overall blood samples collected in the Lazio Region (n = 972), 17 were positive for *Dirofilaria *by modified Knott method, giving a prevalence of 1.75% (95%CI 1.06%–2.85%). Dirofilariosis prevalences observed in each Province were: 13/730 (95%CI 1.78%; 0.99%–3.11%) in Roma, 1/97 (1.03%) in Latina, 2/46 (4.35%) in Rieti, and 1/18 (12.5%) in Viterbo. No one of the 73 animals tested in the Frosinone Province were parasitized.

**Figure 1:**
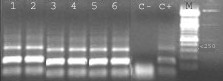
**PCR. **Amplification products with specific primers for *D. repens *shown by 6 positive blood samples (lanes 1–6); negative (7) and positive (8) controls, molecular marker 100 bp (M).

Filarial species identified by microscopy in all positive samples was *D. repens*. Molecular tools confirmed the number of positive and negative dogs and the species identification, as demonstrate by the pattern shown by all positive samples. In fact, the application of primers for "filarial parasites", and the sequencing of amplified products analysed by Clustal, indicated a sequence similarity of 99.8% with *D. repens*. The first identification was then tested by specific primers for *D. repens *and *D. immitis *[21]: all positive samples were amplified only by *D. repens *primers, so confirming the sequence analysis. An example is reported in fig. 1.

**Figure 2:**
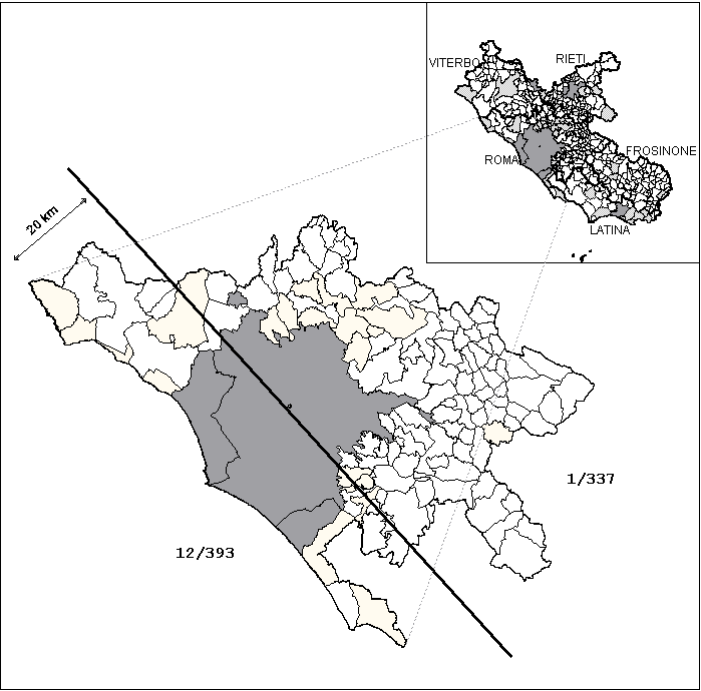
**Dirofilariosis in the Lazio Region **Distribution of dirofilariosis in the Lazio region (in the mainframe). Prevalence data in the Province of Rome respect to the distance to the coast line (< 20 km; > 20 km). In pale grey tested negative municipalities, in dark grey the positive municipalities

The results regarding the Roma province are summarized in tab. 1. A more detailed analysis concerning dogs found positive showed that 12 (among 393) came from coastal areas (9 from extra-urban and 3 from urban territory) and 1 (among 337) from the inner-land (urban territory) (fig. 2). Four positive dogs came from small kennels in the two areas along the coast; the other 9 lived in private houses. All the dogs (n = 91) tested from the largest public kennel within Rome municipality and located in the urban area, which hosts stray dogs coming from the entire city, were negatives.

**Table 1:** Province of Rome: tested dogs (n° positive) on the basis of distance from the coastline, age class and sex.

**distance from the coastline**	**≤ 20 km**			
				
**age class (months)**	**female**	**male**	nd*	**f+m+nd**
				
1(< 13)	10 (0)	12 (0)	-	22 (0)
2 (13–36)	26 (0)	28 (0)	2 (0)	56 (0)
3 (37–120)	56 (1)	75 (5)	2 (1)	133 (7)
4 (> 120)	4 (0)	17 (0)	-	21 (0)
nd*	41 (0)	65 (5)	55 (0)	161(5)
**all age classes coastal**	**137 (1)**	**197 (10)**	**59 (1)**	**393 (12)**
				
	**> 20 km**			
	
1(< 13)	12 (0)	17 (0)	-	29 (0)
2 (13–36)	34 (0)	47 (0)	-	81 (0)
3 (37–120)	68 (0)	73 (0)	-	141 (0)
4 (> 120)	3 (0)	6 (1)	-	9 (1)
nd*	7 (0)	21 (0)	49 (0)	77 (0)
**all age classes inner land**	**124 (0)**	**164 (1)**	**49 (0)**	**337 (1)**
				
**total (coastal + inner land)**	**261 (1)**	**361 (11)**	**108 (1)**	**730 (13)**

*Not determined

Since a large number of dogs were of unknown age (238/730) and 5 of them were positive, the effect of age on the risk for dirofilariosis was not evaluable, although all the remaining positive dogs (n = 8) were > 36 months of age. Even if the number of microfilaraemic dogs was higher in rural environment (9/374; 2.4%) than in urban areas (4/343; 1.2%), no effect of the urbanization level of the origin area was observed at the univariate logistic regression analysis. Similarly no effect was observed with reference neither to breeding in kennel/private house nor to hair length.

On the contrary, the origin from coastal territories (inner 20 km from coast line) represents a significant risk factor for canine dirofilariosis in the Roma Province. In fact, the risk for dirofilariosis was more than 10 fold higher in dogs coming from coastal areas than from the inner-land (OR 10.58; 95%CI 1.37 to 81.82)

Moreover, the risk for dirofilariosis turned out higher in males than in female dogs (OR 8.19; 95%CI 1.04 to 62.50). Nevertheless, at the multivariate analysis the effect of sex was lost, because of the confounding effect of the distance from the coastline. Then, adjusting for the other variables in the multivariate model, dogs more at risk in the Roma Province were the ones living within 20 Km to the coastline (OR 10.43; 95%CI 1.23 to 88.71).

## Discussion

In the present study canine filariosis was detected for the first time in the Lazio Region. Its prevalence is higher along the seacoast territory and, inner land, at the boundary with the Umbria Region. The etiological agent of the infections was *D. repens*, species before reported in the Region only in foxes [18]. The absence of notification of this filarial worm in dogs, in spite of its report in wild animals and in dogs living in the neighbouring Regions [8,10] could be due to the recent passage of the parasite from wild to domestic environment, or could be related to the low pathogenicity of the nematode that usually make difficult, also to trained veterinarians, to suspect the infection, unless during specific survey.

*D. repens *is the species more prevalent also in the canine population of the bordering Tuscany [17], and it is well represented in the adjacent Umbria and Campania Regions, where *D. immitis *and *A. reconditum*, respectively, are the predominant filarial nematodes [8,10]. Infection rates observed, ranging from 1.03 to 12.5%, are in general agreement with those reported in the aforementioned Regions, even if data regarding Provinces other than Roma have to be considered only indicative, because coming from only one kennel per province and few other extra samples.

About the risk factors evidenced, some of the our data fit in with the conclusions of previous studies (no influence of hair length and sex), [4,8,10], whereas the influence of the age of the dog, not evidenced in this study, has been considered by the Authors cited above in a discordant way. However, the low prepatence times observed in experimental infections suggest that higher infection rates in adult individuals are simply related to the longer exposition times. Unfortunately, the typology of the dog population tested (kennels and urban dogs) hampered the investigation on other potential risk factors like the use of the animals (hunting practice?), the presence of a nocturnal shelter and the number of hours spent on open air. Anyway, low infection rates evidenced would have made difficult such an analysis.

The large confidence intervals of the OR at the multivariate analysis is an expression of the uncertainty of the risk association between dirofilariosis and coastal provenience of dogs evidenced in this study. This result is likely due to the low number of positve dogs among the hundreds tested in this survey. However, the higher prevalence of dirofilariosis in dogs living near the coast was already detected [24]. The apparent higher risk for those animals, can't be explained by a different Culicidae fauna, since the known vector species, *Cx. pipiens *and *Ae. albopictus*, are both widespread in the territory studied. The difference should be recognized in climatic conditions, that can favour the fast development of large mosquito population and, over all, can be more suitable for a fast development of larval stages of *D. repens *in mosquitoes and for their transmission [25], as suggested to explain also the higher infection rates by *D. immitis *along river valley [26].

This survey failed to evidence *D. immitis*, *A. reconditum *and *A. dracunculoides*. The absence of *D. immitis *as an autochthonous etiological agent of infection is indirectly confirmed by the results of the post mortem examination of about 550 dogs coming from the province of Roma in the period 2001–2003, regularly inspected in the hearth cavities, which proved all negative (Pathology Department of the Istituto Zooprofilattico Sperimentale, pers. comm.). Nevertheless, recent studies showed that canine filariosis by both species is increasing its geographic spread. In particular, the evident progression along river valleys recorded in the Umbria Region [8] and confirmed by the moderate prevalence of dirofilariosis detected in our study in the Rieti and Viterbo provinces, bordering to that region, is a threat of an imminent introduction of cardiopulmonary dirofilariosis also in the Lazio Region and in the Roma territory. In fact, even if laboratory data suggested that infection by *D. repens *may play a protective role against infection by *D. immitis*, so "defending" the dog from more pathogenic species [27], the recent report of *D. immitis *in mosquitoes of the Region caught in the urbanized area [28] is challenging.

Dogs of the Region turned out also *A*. *reconditum *and *A. dracunculoides *free. The first species, largely present in the adjacent Regions, and the second, observed in foxes of the province of Roma [29], were undetected by microscopy and "filarial" primers during this survey. Their absence could be explained by the sampled dog population, practically lacking of hunting and shepherd dogs, probably more exposed to the arthropod vectors.

Finally, the survey carried out, reporting the presence of the slightly pathogen *D. repens *in the dog population, reassures on the sanitary status of the animals but, at the same time, is alarming for humans that could be infected by zoo-antropophilic vector mosquitoes like *C. pipiens *and *Ae. albopictus*. In fact, *D. repens *is the species to date recognized in human infections reported in Italy. The zoonotic impact of this parasitic infection has been recently evaluated [30]: more than one hundred of human infections have been reported in 5 years from the whole country. Therefore, a specific education program in preventing and detecting the disease in dogs as well as in humans, for citizen, doctors and veterinary, has to be encouraged.

## Competing interests

The author(s) declare that they have no competing interests.

## Authors' contributions

PS partecipated at the design of the study and drafted the manuscript. SG carried out the molecular tests. MDP contribute to acquire the samples and to carry out microscopic examinations. MS designed the sampling and performed the statistical analysis. FS coordinated the working group and revised critically the manuscript. GC helped to draft the manuscript and revised it. All authors read and approved the final manuscript.

## Pre-publication history

The pre-publication history for this paper can be accessed here:


